# Case Report: A Programmed Cell Death-1 Inhibitor-Related Abdominal Fibroinflammatory Reaction Affecting Multiple Organs in A Non-Small-Cell Lung Cancer Patient

**DOI:** 10.3389/fimmu.2022.874932

**Published:** 2022-07-01

**Authors:** An-Tian Chen, Yue-Quan Shi, Bei Tan, Liang Zhu, Ya-Ping Luo, Wei Zhong, Meng-Zhao Wang, Yan Xu

**Affiliations:** ^1^Department of Cardiology, Peking Union Medical College Hospital, Chinese Academy of Medical Sciences and Peking Union Medical College, Beijing, China; ^2^Department of Respiratory and Critical Care Medicine, Peking Union Medical College Hospital, Chinese Academy of Medical Sciences and Peking Union Medical College, Beijing, China; ^3^Department of Gastroenterology, Peking Union Medical College Hospital, Chinese Academy of Medical Science and Peking Union Medical College, Beijing, China; ^4^Department of Radiology, Peking Union Medical College Hospital, Chinese Academy of Medical Science and Peking Union Medical College, Beijing, China; ^5^Department of Nuclear Medicine, Peking Union Medical College Hospital, Chinese Academy of Medical Science and Peking Union Medical College, Beijing, China

**Keywords:** pembrolizumab, non-small-cell lung cancer, immune-related adverse events, fibroinflammatory reaction, immunothearpy

## Abstract

Immunotherapy utilizing programmed cell death-1 (PD-1)/PD-L1 inhibitors has been regarded as a rising hope for tumor patients, and their effects have been demonstrated in many clinical trials. However, immune-related adverse events also occur in patients and can sometimes have severe consequences. Pembrolizumab (Keytruda) is a humanized monoclonal anti-PD-1 antibody that has been approved by the US Food and Drug Administration for non-small-cell lung cancer. Here, we report a rare case of an abdominal fibroinflammatory reaction that affected multiple organs during anti-PD-1 immunotherapy using pembrolizumab in a non-small-cell lung cancer patient. The patient’s case demonstrates that immunotherapy-related abdominal fibroinflammatory reactions need to be considered, especially for patients with a history of pre-existing conditions in the abdomen. Glucocorticoids may be useful as a treatment when a diagnosis is confirmed.

## Introduction

Immunotherapy has developed into a treatment option for patients with non-small-cell lung cancer (NSCLC) (1) and is regarded as a successful trend in NSCLC treatment (2). However, every successful therapy has deficiencies, and immune-related adverse events (irAEs) are observed during immunotherapy treatments. In patients using pembrolizumab, up to 60% suffered from adverse events, and less than 10% had grade 3/4 toxicities (3). IrAEs can occur in different organs with a variety of clinical manifestations. Some irAEs have phenotypes that mimic inflammatory diseases, including inflammatory arthritis, myositis, and vasculitis. Although rare, immune checkpoint inhibitor (ICI)-related fibroinflammatory diseases have been reported. Programmed cell death-1 (PD-1) inhibitor-related sclerosing cholangitis has been reported to have clinical features of biliary dilation, diffuse thickening of the extrahepatic biliary tract with or without multiple strictures of the intrahepatic biliary tract, and normal serum immunoglobulin G4 (IgG4) (4). Systemic sclerosis, characterized by progressive fibrosis of the skin and internal organs, has been reported following treatment using ICIs (5). Retroperitoneal fibrosis, a rare disease characterized by fibrotic tissue in the retroperitoneum (6), rarely occurs secondary to anti-PD-1 therapy in patients with malignant tumors (7, 8). This phenomenon requires further insights and discovery. Here, we report a rare condition of an abdominal fibroinflammatory reaction affecting multiple organs following immunotherapy with pembrolizumab.

## Case Description

A 66-year-old female non-smoker presented with shortness of breath in May 2019 and was admitted to a local hospital. Chest CT revealed a spiculated mass in the left upper lobe, multiple pulmonary nodules, mediastinal lymphadenopathy, and pericardial effusion. Lung adenocarcinoma was diagnosed through a bronchoscopic biopsy. Molecular testing detected a p.L858R mutation in epidermal growth factor (EGFR) exon 21. Multiple metastases were confirmed in the lung, liver, pericardium, bone, brain, and distant lymph nodes. The tumor stage reached cT4N3M1c (stage IV B). She was prescribed gefitinib and had a progression-free period of 7 months. She was then switched to osimertinib treatment because EGFR exon 20 p.T790M (+) was detected in the progressive pleural fluid. However, four months later, the disease was characterized by progressive bone and brain metastases. The patient’s past history indicated that she had suffered intestinal obstruction without clear cause, and the abdominal surgery was compelled to perform at age 13, while the intestinal obstruction recurred twice around 2003 and 2006.

**Figure 1:**
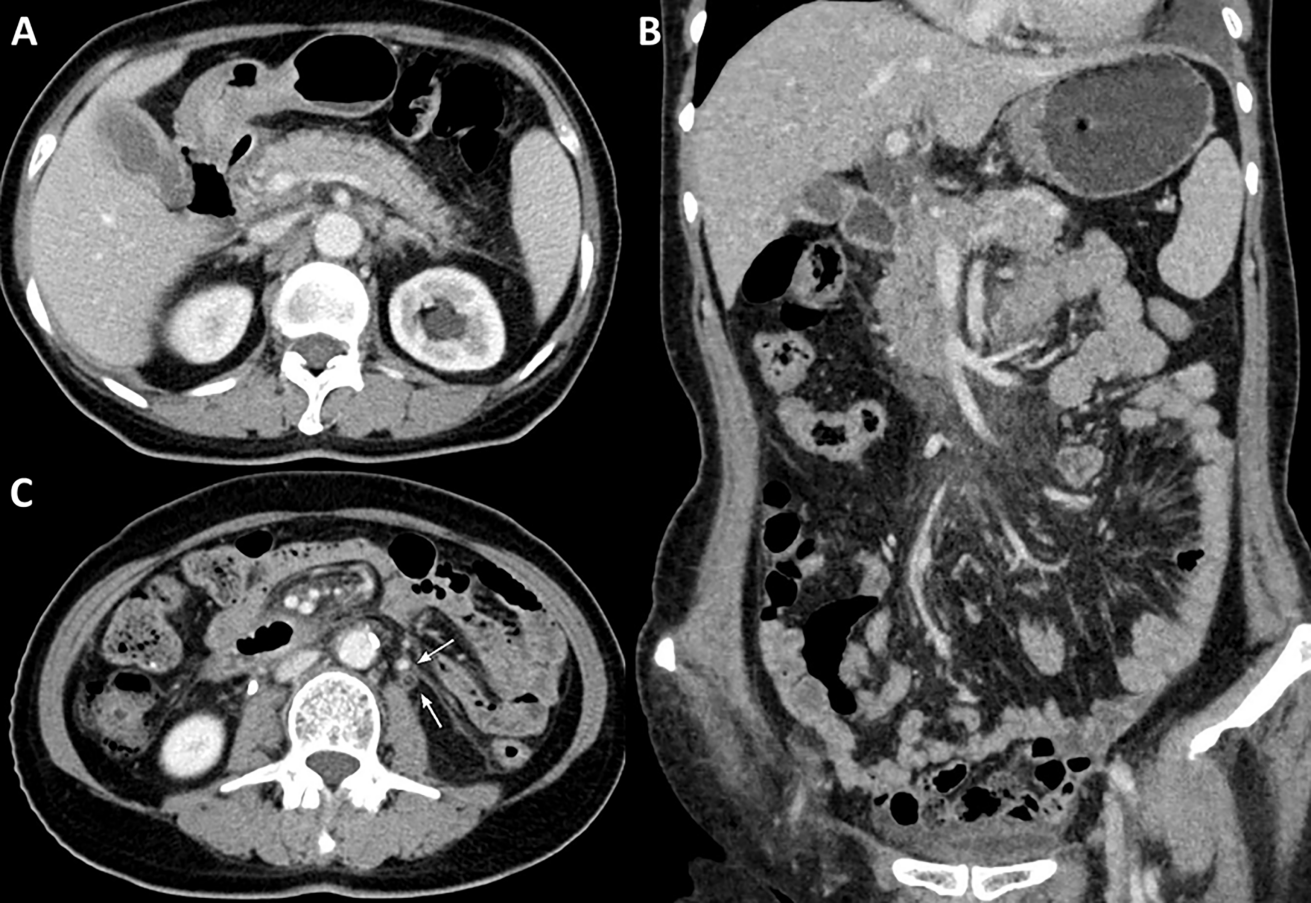
Contrast-enhanced abdominal CT. **(A)** Contrast-enhanced CT reveals exudation around the pancreas and slight dilatation of the pancreatic duct, **(B)** a thickened peritoneum and cloudy mesentery, and **(C)** avid enhancement with accompanying ureterectasia (white arrow).

**Figure 2:**
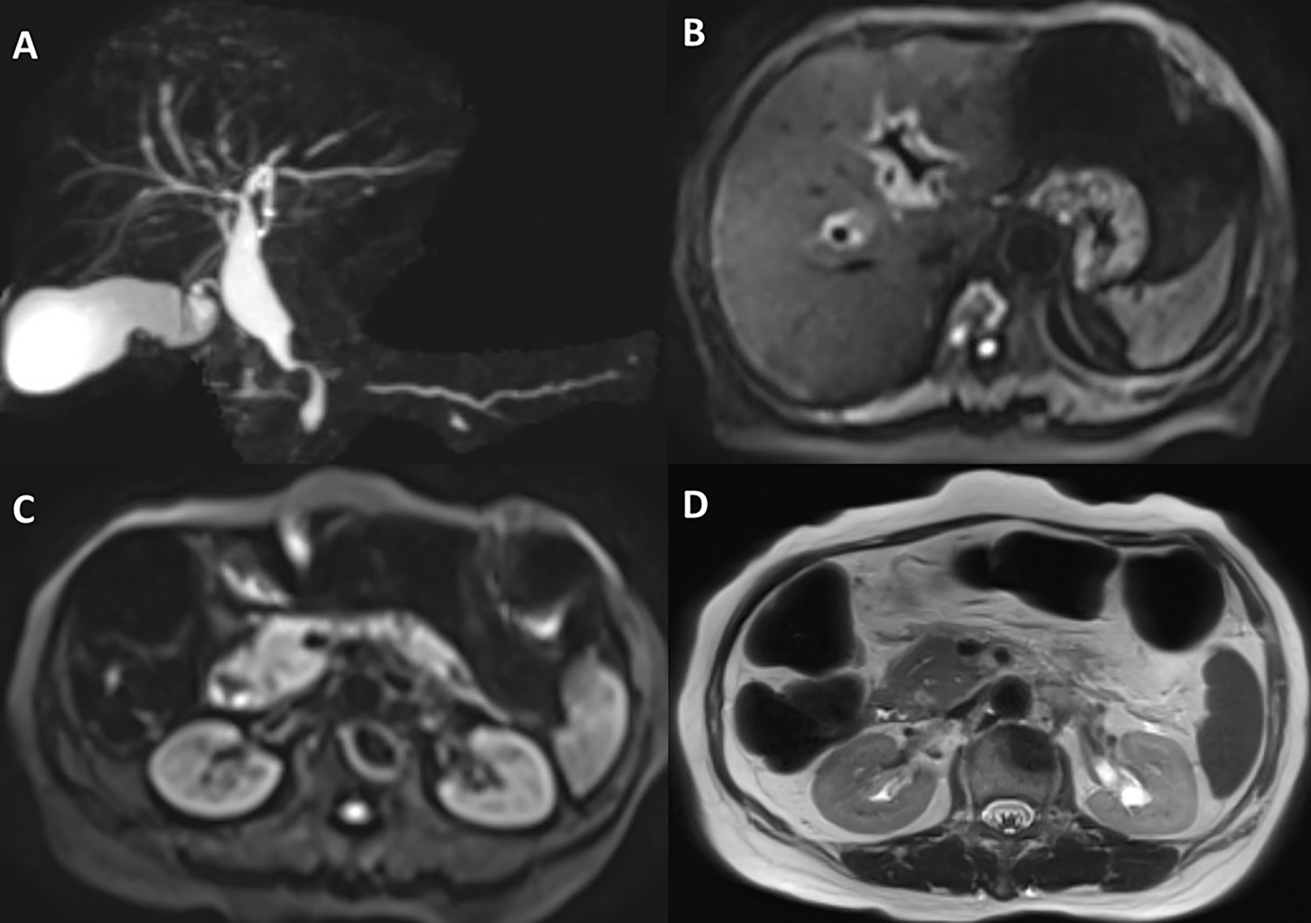
Magnetic resonance cholangiopancreatography and abdominal magnetic resonance imaging. **(A)** Magnetic resonance cholangiopancreatography shows highly suspected diffuse hypertrophy of the extrahepatic biliary tract, inferior biliary tract stenosis, and multiple strictures of the intrahepatic biliary tract. **(B)** Abdominal magnetic resonance imaging suggests that the intrahepatic bile duct wall is significantly thickened, and diffusion was significantly restricted on diffusion-weighted imaging. **(C)** Diffusion-weighted imaging reveals that the entire pancreas demonstrated diffuse high signal. **(D)** A thickened peritoneum is evident.

**Figure 3:**
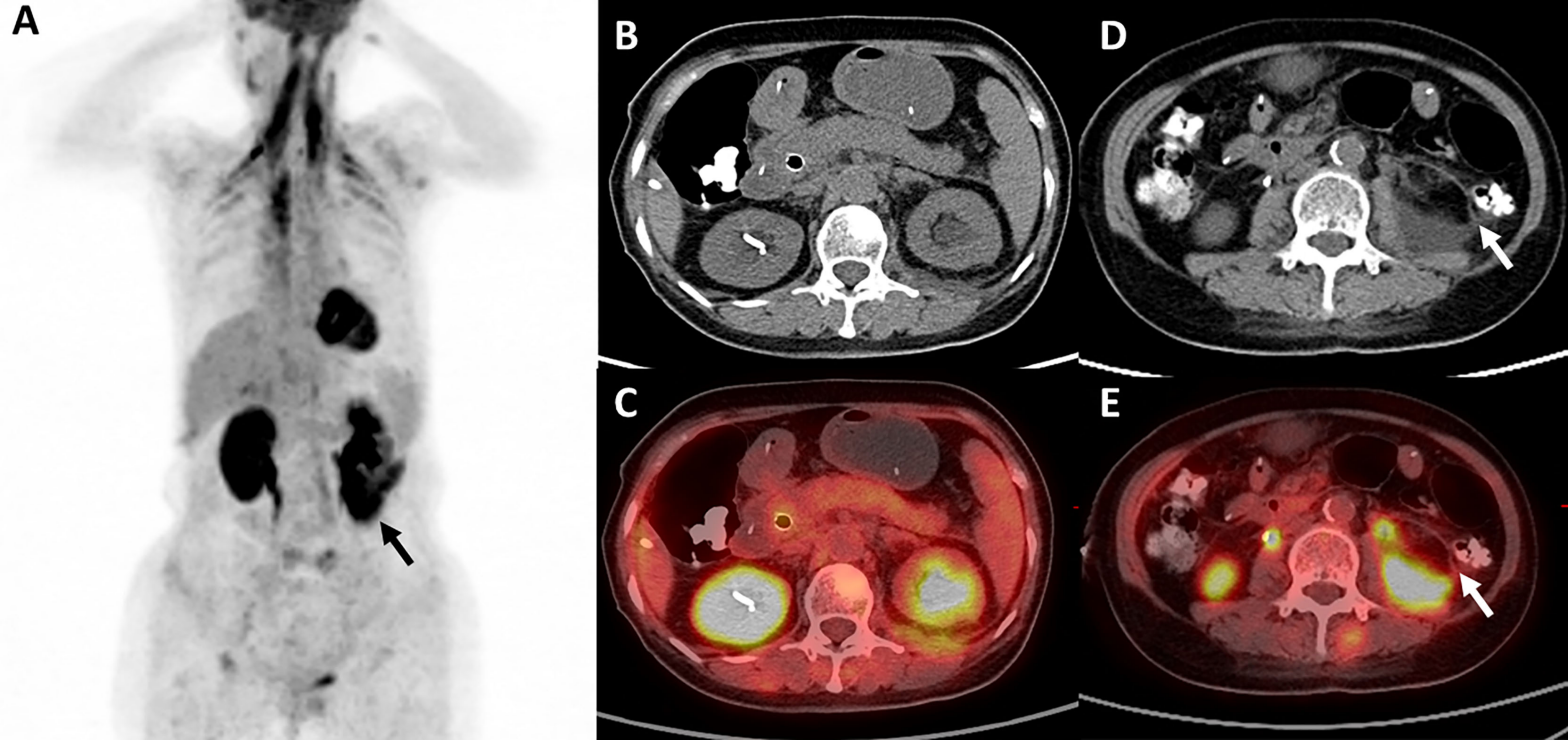
^18F^FDG positron emission tomography-computed tomography. **(A, D, E)** ^18F^FDG PET-CT reveals left renal perirenal fascial thickening with abnormal metabolic elevation (black arrow and white arrow), and **(B, C)** inflammatory change in the pancreas.

The patient received four cycles of pemetrexed, carboplatin, bevacizumab, and pembrolizumab starting in August 2020. Two cycles of pemetrexed and pembrolizumab were administered as maintenance therapy. Stable disease (SD) was achieved and persisted after 2 cycles ICIs combined treatment. Four months after the first use of pembrolizumab, the patient experienced abdominal pain caused by right ureterectasia. A double-J stent was used to relieve her symptoms. One month later, the patient experienced recurrent abdominal pain. Laboratory tests showed that her total bilirubin rose from 8.9 μmol/L to 54.2 μmol/L (normal: 5.1–22.2 μmol/L), direct bilirubin rose from 3 μmol/L to 45.9 μmol/L (normal: ≤6.8 μmol/L), alanine serum aminotransferase rose from 103 to 162 U/L (normal: 7–40 U/L), aspartate serum aminotransferase rose from 92 to 446 U/L (normal: 13–35 U/L), and gamma-glutamyl transpeptidase rose from 488 to 1,350 U/L (normal: 7–45 U/L). Laboratory testing also revealed elevated serum amylase (132 U/L, normal: 35–115 U/L) and lipase (746 U/L, normal: 2–53 U/L). Contrast-enhanced CT revealed exudation around the pancreas, slight dilatation of the pancreatic duct (**Figure 1A**), a thickened peritoneum, and cloudy mesentery (**Figure 1B**), which are encountered often in abdominal inflammatory reactions such as acute pancreatitis or lupus. An unexpected finding, however, was that the left ureter was thickened and showed avid enhancement (**Figure 1C**) accompanied by ureterectasia (**Figure 1A**). Magnetic resonance cholangiopancreatography (MRCP) showed narrowing of the distal bile duct and dilatation of the extrahepatic bile duct beyond the pancreatic portion. Instead of proportional dilatation in continuation with the extrahepatic bile duct, the intrahepatic duct showed multiple strictures (**Figure 2A**). The narrowed segments of the biliary tracts were thickened symmetrically, and diffusion was significantly restricted on diffusion-weighted imaging (**Figure 2B**). Segmental stenosis of the main pancreatic duct was noted in the pancreatic head, with slight dilation of the upstream pancreatic duct, which did not exceed 3 mm (**Figure 2A**). The entire pancreas demonstrated diffuse high signal on diffusion-weighted imaging (**Figure 2C**). A thickened mesentery and peritoneal fascia were also observed (**Figure 2D**). However, there was no evidence of pancreatic metastases or peritoneal seeding. In addition, obstruction of the common bile duct was confirmed, but endoscopic retrograde cholangiopancreatography failed because of severe stenosis. Percutaneous transhepatic cholangiography and drainage and biliary stent implantation was performed for obstructive cholangitis considering her continuously elevated bilirubin level. ^18F^FDG positron emission tomography-computed tomography (PET-CT) (**Figure 3**) detected left renal perirenal fascial thickening with abnormal metabolic elevation and inflammatory changes in the bile duct and pancreas, without evidence of abdominal tumor-metastasis. Laboratory tests showed that the IgG4 level was within normal limits (IgG4: 475 mg/L, normal: 80–1400 mg/L), anti-nuclear antibodies were positive with a low titer of 1/80, and anti-neutrophil cytoplasmic antibodies were negative. Meanwhile, cytokines analysis showed that the interleukin 6 (IL-6) (IL-6: 8.4 pg/mL, normal: <5.9 pg/mL)and tumor necrosis factor-alpha (TNFα) (TNFα 8.9 pg/mL, normal: <8.1 pg/mL) were slightly elevated, while IL-8 and IL-10 were within normal limits.

**Figure 4:**
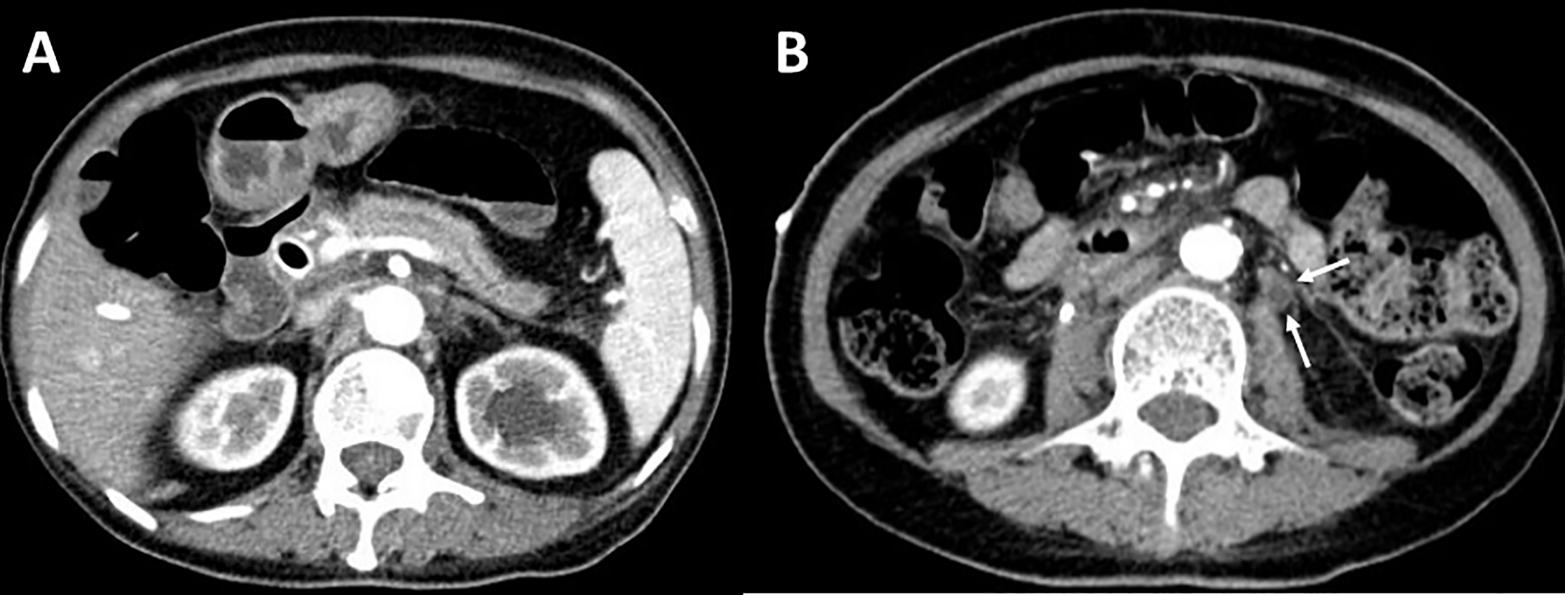
Contrast-enhanced abdominal CT after treatment. **(A)** Contrast-enhanced CT reveals less inflammatory reactions of the pancreas. **(B)** Thickness of peritoneum maintained without further thickening and left ureterectasia partly relieved (white arrow).

Overall, the patient suffered from bile system disease, bilateral ureteral obstruction, chronic pancreatitis, and a thickened peritoneum and bilateral perirenal fascia. An abdominal fibroinflammatory reaction was highly suspected, which may have been caused by ICI treatment. The patient was treated with glucocorticoids (methylprednisolone 40mg daily, 0.8mg/kg), and her symptoms were well-controlled without aggravation. The patient was followed up for 6 months, and an enhanced abdominal CT revealed that there was less inflammatory reaction of the pancreas (**Figure 4A**), and the thickness of the renal fascia and peritoneum maintained without further thickening (**Figure 4B**). More importantly, her ureterectasia on the left side was partly relieved (**Figure 4B**), and a double-J stent was not needed.

## Discussion

Our patient presented with bilateral ureterectasia, bile system disease, chronic pancreatitis, and a thickened peritoneum, mesentery, and bilateral perirenal fascia. After excluding other possible causes, the ICI treatment history, imaging, and steroid-sensitive response suggested an abdominal fibroinflammatory reaction elicited by the anti-PD-1 therapy.

Immunotherapy-related events can be diagnosed only through comprehensive analysis and the elimination of other possible causes. First, it was the discovery of the thickening and hypermetabolic foci of the abdominal peritoneum and multiple organs that helped identify an extensive fibroinflammatory reaction in different organs and tissues. The thickened peritoneum detected in the radiological findings on abdominal CT also suggested an abdominal inflammatory reaction. The abdominal MRI and MRCP imaging patterns highly resembled sclerosing cholangitis and autoimmune pancreatitis (9, 10). Second, the diagnosis of the tumor made it necessary to exclude abdominal changes caused by metastasis. The results of ^18F^FDG PET-CT and MRI did not support abdominal tumor-metastasis-related conditions. Third, the clinical manifestations in this patient were consistent with IgG4-related disease, which is an immune-mediated fibroinflammatory disease, but it did not meet the diagnostic criteria for IgG4-related disease because a normal serum IgG4 concentration was detected. Fourth, immunotherapy-related sclerosing cholangitis, chronic pancreatitis, or retroperitoneal fibrosis can occur after immunotherapy, which may be an unusual adverse reaction and an exclusionary diagnosis. Ultimately, the diagnosis of an immunotherapy-related abdominal fibroinflammatory reaction was confirmed when the patient’s symptoms were well-controlled after steroid treatment.

Fibroinflammatory disorders can occur in a spectrum of diseases. Retroperitoneal fibrosis is a rare disease characterized by chronic inflammation and profound fibrosis in the peri-aortic and peri-iliac organs or tissues (11). Chronic pancreatitis is characterized by a pathological fibroinflammatory syndrome of the pancreas (12). Sclerosing cholangitis is characterized by inflammation and fibrosis of the bile ducts and the liver (13). The IgG4-related disease is a systemic fibroinflammatory condition that affects multiple organs (14). Scleroderma occurs because of vascular disorders and fibrosis due to genetic and molecular changes (15). Until now, sclerosing cholangitis, retroperitoneal fibrosis, and chronic pancreatitis have been reported to be induced by ICI treatment; however, rare cases of extensive fibroinflammatory disorders in specific individuals have been reported.

The mechanism of fibroinflammatory reaction as an irAE is not fully understood, but there are potential mechanisms. Fibroinflammatory disorders are caused by a combination of genetic and environmental factors. Retroperitoneal fibrosis secondary to immunotherapy may be caused by the rejuvenation of antigen presentation by antigen-presenting cells (16). The mechanism underlying sclerosing cholangitis is still not fully understood. Tissues of pancreatitis and sclerosing cholangitis tissues share dominantly CD8+ cells in a CD3+ T cells infiltrate pathologically (17). Scleroderma can also be induced by immunotherapy. T cells are thought to respond more actively after ICI treatment, which results in not only the desired treatment effects but also the production of self-reactive T cells (18). Previously existing skin damage, such as that caused by ultraviolet light, could lead to exposure of self-antigens and thereby enhance T cell generation (19).

Underlying mechanism of fibroinflammatory reaction should be further explored. When stimulated by certain antigens, B cells proliferate; some differentiate into plasma cells, and IgG is produced. However, in some cases, IgG4 production is preferred (20). The IgG4 subclass of antibodies is expressed in alternative Th2 environments characterized by high levels of IL-10 (21). Furthermore, when an enhanced Th2 signal is observed, cytokines related to Th2 signaling, such as IL-10 and IL-13 are found to be elevated (22). It is known that Th2-skewed responses favor fibrocyte differentiation. Therefore, Th2 contributes to fibrotic effects. In the treatment of malignant tumors, Th2 cells may have anti-tumor potential by modulating the tumor microenvironment (23). PD-1 blockade is believed to inhibit Th2 response (24). However, the Th2 pathway exhibits an enhancement during follow-up with increasing levels of cytokines such as IL-4 and IL-5 (25). In addition, Th2 pathways may be implicated in patients with irAEs (25). For example, the serum level of IL-6 is increased in nivolumab-associated psoriasiform dermatitis, which indicates the involvement of Th2 pathways (26). It may also induce chronic pancreatitis since IL-6 is associated with exocrine pancreatic diseases including chronic pancreatitis and its serum level in chronic pancreatitis patients was found with a remarkable elevation compared to control subjects (27). Thus, it is reasonable to propose a possible mechanism of abdominal fibroinflammatory reaction as an irAE in immunotherapy. Although Th2 pathways are often suppressed in immunotherapy, related pathways might recover during follow-up and cause irAEs such as psoriasiform dermatitis.

Similarly, in this case, considering the patient’s history of recurrent intestinal obstruction and an abdominal operation, the exudation of abdominal inflammatory lesions might have been aggravated after ICI treatment. To be specific, past abdominal inflammatory injuries expose self-antigen, which will enhance functions of T cells. At the same time, treatment effects of immunotherapy as well as APCs rejuvenated by antigen presentation would promote T cells. Activation of CD4/CD8 will start and lead to generation of follicular helper T (Tfh) cells, which could promote plasmablast differentiation (28). In fact, PD-1, which is surface receptor on Tfh cells, is responsible for negatively regulation (29). Thus, it is reasonable to suggest such process in activating plasma cells and T cells and progressively causing fibroinflammatory disorders. In summary, a pre-existing inflammatory state in the abdomen may accelerate the entire process and lead to an abdominal fibroinflammatory reaction being eventually triggered by immunotherapy.

There are some limitations. The patient did not have PD-L1 IHC test before or after osimertinib treatment, thus we do not know the PD-L1 expression situation and whether there was any change or not. Also, though abdominal fibroinflammatory reaction was obvious and typical in radiological examinations, it was hard to performed abdominal inflammatory lesions biopsy for pathological examinations.

In summary, we reported a rare condition of an abdominal fibroinflammatory reaction that affected multiple organs following immunotherapy with pembrolizumab, which is barely known and unfamiliar in clinical practice. Immunotherapy-related abdominal fibroinflammatory reactions need to be considered, especially for patients with a history of celiac disease. Glucocorticoids may be useful as a treatment when a diagnosis is reached.

## Data Availability Statement

The original contributions presented in this study are included in the article/supplementary material. Further inquiries can be directed to the corresponding author.

## Ethics Statement

Written informed consent was obtained from the individual(s) for the publication of any potentially identifiable images or data included in this article.

## Author Contributions

ATC wrote the manuscript. YX designed the study and revised the manuscript. All authors contributed to the manuscript and approved the submitted version.

## Funding

This study was supported by the Youth Program of the National Natural Science Foundation of China (to YX) (grant number: 82003309) and by CAMS Innovation Fund for Medical Sciences (CIFMS) (to YX) (grant number: 2021-I2M-C&T-B-014).

## Conflict of Interest

The authors declare that the research was conducted in the absence of any commercial or financial relationships that could be construed as a potential conflict of interest.

## Publisher’s Note

All claims expressed in this article are solely those of the authors and do not necessarily represent those of their affiliated organizations, or those of the publisher, the editors and the reviewers. Any product that may be evaluated in this article, or claim that may be made by its manufacturer, is not guaranteed or endorsed by the publisher.
